# Predicting Minute Ventilation from Heart Rate in Adolescents: A Tool for Environmental Health Studies

**DOI:** 10.3390/environments12120448

**Published:** 2025-11-21

**Authors:** Celia Cacho, Meghana Giri, Kyung Hwa Jung, Ruskin Del Mundo, Aimee Layton, Stephanie Lovinsky-Desir

**Affiliations:** 1 Vagelos College of Physicians and Surgeons, Columbia University, 630 W 168th St, New York, NY 10032, USA; 2 Division of Pediatric Pulmonology, Department of Pediatrics, Vagelos College of Physicians and Surgeons, Columbia University, 3959 Broadway CHC-745, New York, NY 10032, USA; 3 Division of Pediatric Cardiology, Department of Pediatrics, College of Physicians and Surgeons, Columbia University, 630 West 168th St, New York, NY 10032, USA;; 4 Department of Environmental Health Sciences, Mailman School of Public Health, Columbia University, 722 West 168th St, New York, NY 10032, USA

**Keywords:** minute ventilation, adolescents, air pollution, environmental health, predictive modeling, inhaled pollution dose

## Abstract

Minute ventilation (VE) is central to understanding the interplay between air pollution and exercise. However, real-time measurement of VE in environmental health research is often limited by access to equipment and technical expertise. We aimed to (1) develop predictive equations for VE based on heart rate (HR) in adolescents using metabolic exercise testing data, (2) evaluate which demographic factors influenced model accuracy, and (3) compare our equations to previously published equations applied to our sample. We analyzed cardiopulmonary exercise test (CPET) data from 41 patients. VE was log-transformed, and generalized estimating equations (GEE) were used to model associations between HR and VE, adjusting for age, sex, race, ethnicity, and BMI. In the fully adjusted model, HR was a strong predictor of VE (*p*-value < 0.001); only sex was a significant covariate (*p* = 0.003). Stratification revealed a higher predicted VE at a given HR for males compared to females (y_male_ = 0.020x + 0.813 vs. y_female_ = 0.019x + 0.708, where y = lnVE and x = HR) with a pseudo-R^2^ of 0.80 for males and pseudo-R^2^ of 0.82 for females. Our predictive equations had the lowest average percent difference between measured and predicted VE, whereas prior models under- or overestimated VE in our sample. Overall, sex-specific GEEs provide a practical method to estimate VE from HR in adolescents and can serve as tools to support exposure assessment and future applications in environmental health research.

## 1. Introduction

Although the harmful effects of air pollution on lung development and overall health are well-established, precise quantification of an individual’s daily inhaled dose remains challenging and critical for understanding variability in exposure and downstream health outcomes [1]. This dose is determined by not only ambient pollutant concentrations but also the volume of air inhaled during daily activities [2]. Minute ventilation (VE), defined as the volume of air inhaled into or exhaled from the lungs per minute, reflects the integrated demands of the respiratory and cardiovascular systems at rest and during activity. In a study by Daigle et al., a 3.3-fold increase in minute ventilation led to a more than 4.5-fold increase in total ultrafine particle deposition in the lungs [3]. Because VE varies substantially with activity level, precise quantification is essential for assessing cardiopulmonary function and for estimating differential exposure to pollution. This variability helps explain why people living in the same environments may experience different exposure doses and, consequently, different health outcomes.

Direct measurement of VE, however, requires cardiopulmonary exercise testing (CPET), which is costly, time-intensive, and typically limited to controlled laboratory settings [4]. These logistical demands restrict participation to individuals with access to well-resourced academic centers, resulting in the systematic underrepresentation of many populations—including adolescents—in environmental health research. Adolescence represents a particularly critical yet understudied period for such work, as this developmental stage is characterized by rapid physiologic changes, evolving activity patterns, and heightened vulnerability to environmental stressors that can influence long-term respiratory health [1].

To address these limitations, surrogate markers such as heart rate (HR) are often used as proxies for VE in environmental health research [2,5,6]. Although wearable devices capable of estimating respiratory parameters continue to advance, persistent challenges with accuracy, calibration, cost, and comfort limit their feasibility for large-scale research [7]. In contrast, HR monitoring is accessible, noninvasive, and easily integrated into real-world settings, establishing HR as a practical and scalable alternative for estimating VE [8,9]. Developing a validated model that links HR to VE therefore offers a practical approach for advancing both clinical and epidemiologic research.

Existing studies in adults have established the feasibility of linking HR to VE through CPET-derived data. Cruz et al. demonstrated strong linear relationships between HR and VE and highlighted the potential of HR as a practical surrogate for ventilatory demand [6]. Importantly, some adult studies have also observed sex-related differences in the HR–VE relationship, suggesting that biological factors may influence predictive accuracy [5,10]. While these studies provide valuable HR-based predictions of VE, they have been conducted primarily in adults, leaving limited evidence in adolescents.

The objectives of this study were to (1) derive a predictive equation of VE based on HR, a variable that can be easily acquired in field studies for broad application in both clinical and epidemiologic contexts, (2) examine whether HR-based predictions of VE differ by demographic characteristics, and (3) compare the accuracy of our HR–based predictive model with previously published equations when applied to our sample of patients.

## 2. Materials and Methods

We have reported our methods according to the checklist by Hesse et al. for standardized reporting across the field to improve methodological rigor in gas exchange data handling [11].

### 2.1. Study Population

We retrospectively analyzed data from a sample of patients aged 14–18 evaluated in our pediatric cardiopulmonary exercise laboratory. Demographic data were collected from electronic medical records and included age, height, weight, body mass index (BMI), and sex assigned at birth. Most patients were recruited from New York City (Northern Manhattan, Bronx, Brooklyn), Westchester County, Long Island, New Jersey, and Connecticut.

Data were collected from 1 January 2023 to 31 July 2024. Data were extracted from the metabolic cart software system Med Graphics (MGC Diagnostics, Saint Paul, MN, USA) and Vyaire Sentry Suite version 3.0.197 (Jaeger Medical GmbH, Hoechberg, Germany). Patients were referred for CPETs due to respiratory symptoms with exercise, including dyspnea, chest pain, asthma, or palpitations, but the patients in our selected sample demonstrated normal tests as determined by the interpreting team.

### 2.2. Laboratory Cardiopulmonary Exercise Testing

All exercise testing was performed using Med Graphics (MGC Diagnostics, Saint Paul, MN, USA), Mortara electrocardiogram (ECG) system (Hill-Rum Corp., Deerfield, IL, USA), Vyaire Sentry Suite (Jaeger Medical GmbH, Hoechberg, Germany) and Cardiosoft ECG (GE Corp., Boston, MA, USA). Calibrations were performed in accordance with manufacturer guidelines and involved flow sensor calibration prior to each test for the Vyaire equipment and daily for the Med Graphics. Gas calibrations were performed prior to each test for both types of systems. Sampling rates were breath-by-breath with data averaging of every 10 s.

The exercise protocol involved at least 2 min of resting data collection, followed by a standard staged Bruce protocol [12]. Exercise was terminated at the patient’s volition or if a contraindication to continued exercise was met as per standardized guidelines for pediatric exercise testing [13]. A valid test was defined as the patient having achieved a respiratory exchange ratio (RER) of at least 1.1 and a heart rate of at least 80% of predicted maximum. Once peak exercise was achieved, the patients performed 2 min of active recovery and at least 3 additional minutes of passive recovery.

The variables collected from the CPET were as follows: minute ventilation (VE), tidal volume (V_T_), respiratory rate, oxygen consumption (VO_2_), carbon dioxide output (CO_2_), heart rate (HR), RER, blood pressure, end tidal CO_2_, ventilatory equivalent for oxygen and carbon dioxide (Ve/VO_2_ and Ve/VCO_2_) and oxyhemoglobin saturation (SpO_2_).

### 2.3. Statistical Analysis

#### 2.3.1. HR-VE Model Development

We limited our analysis to include CPET data corresponding to pre-start, start exercise, and anaerobic threshold periods, with values averaged over 8 breaths. Values were averaged over 8 breaths to ensure consistent quality across participant data, a method described in a study by Buehler et al. [14]. As a part of data quality assurance, we defined outliers in VE and HR as data points with a >20% decrease relative to the subsequent measurement, occurring either during the transition from pre-start to start exercise or within the same phase. This threshold for outlier exclusion was applied to identify physiologically implausible instances in which VE decreased despite an increase in heart rate, based on data review and expert consensus within the study team. We excluded these outliers (3.1%) from the analysis, as VE and HR are expected to increase during the incremental stage of exercise. The final model included 1637 paired HR–VE observations collected from all patients.

We used multivariable linear regression in generalized estimating equation (GEE) models, with robust standard errors to assess associations between VE and HR. The variable minute ventilation at body temperature and ambient pressure, saturated with water vapor (VE BTPS), a standard VE measurement in CPET, was used as the outcome. VE BTPS was naturally log-transformed, consistent with a previous study showing that an exponential equation provides a more accurate fit than a quadratic model, particularly at low intensity exercise [6]. Model assumptions were evaluated by inspecting residual plots for linearity, homoscedasticity, and normality and no major violations were observed. We began our model selection process with a fully adjusted model including age, sex, race, ethnicity, and BMI as covariates to determine if any of these variables were significant predictors of VE. Sex was the only covariate that was statistically significant in our model; therefore, we presented a more parsimonious set of models stratified by sex, excluding the other insignificant covariates. Lastly, we examined whether the association between VE and HR was modified by sex. A multiplicative interaction term (HR × sex) was included in the interaction model. Coefficients of HR (slope) and intercepts are presented in the following equation: LnVE BTPS = slope × HR + intercept. Because traditional R^2^ is not directly applicable to GEE, model fit was evaluated using a pseudo-R^2^, estimated as the squared Pearson correlation between observed and model-predicted VE values for male and female strata. The *p*-value was set at 0.05.

#### 2.3.2. Comparative Analysis

We compared our sex-stratified HR-VE equation with other similar equations described in the literature to determine whether other models’ equations could accurately predict our patient sample’s VE. First, we calculated the average percent difference between predicted vs. measured VE using our equations. Second, we applied published sex-stratified exponential HR-VE equations to our dataset and calculated the average percent difference between predicted and measured VE. All analyses were performed using SPSS version 26 (Chicago, IL, USA).

## 3. Results

Demographic characteristics for the 41 patients with normal CPET values who were included in the study are reported in Table 1. The majority were male (65.9%), while females accounted for 34.1% of the sample. Regarding race, 39.0% identified as White, 7.3% as Black, 22.0% as Other, and 31.7% declined to report their race. In terms of ethnicity, 19.5% identified as Hispanic, 51.2% as non-Hispanic, and 29.3% declined to disclose their ethnicity. The mean age of participants was 15.4 ± 1.2 years. Average anthropometric measures were as follows: height 165.9 ± 8.8 cm, weight 60.6 ± 10.4 kg, and BMI 22.0 ± 2.5 kg/m^2^.

**Figure 1.**
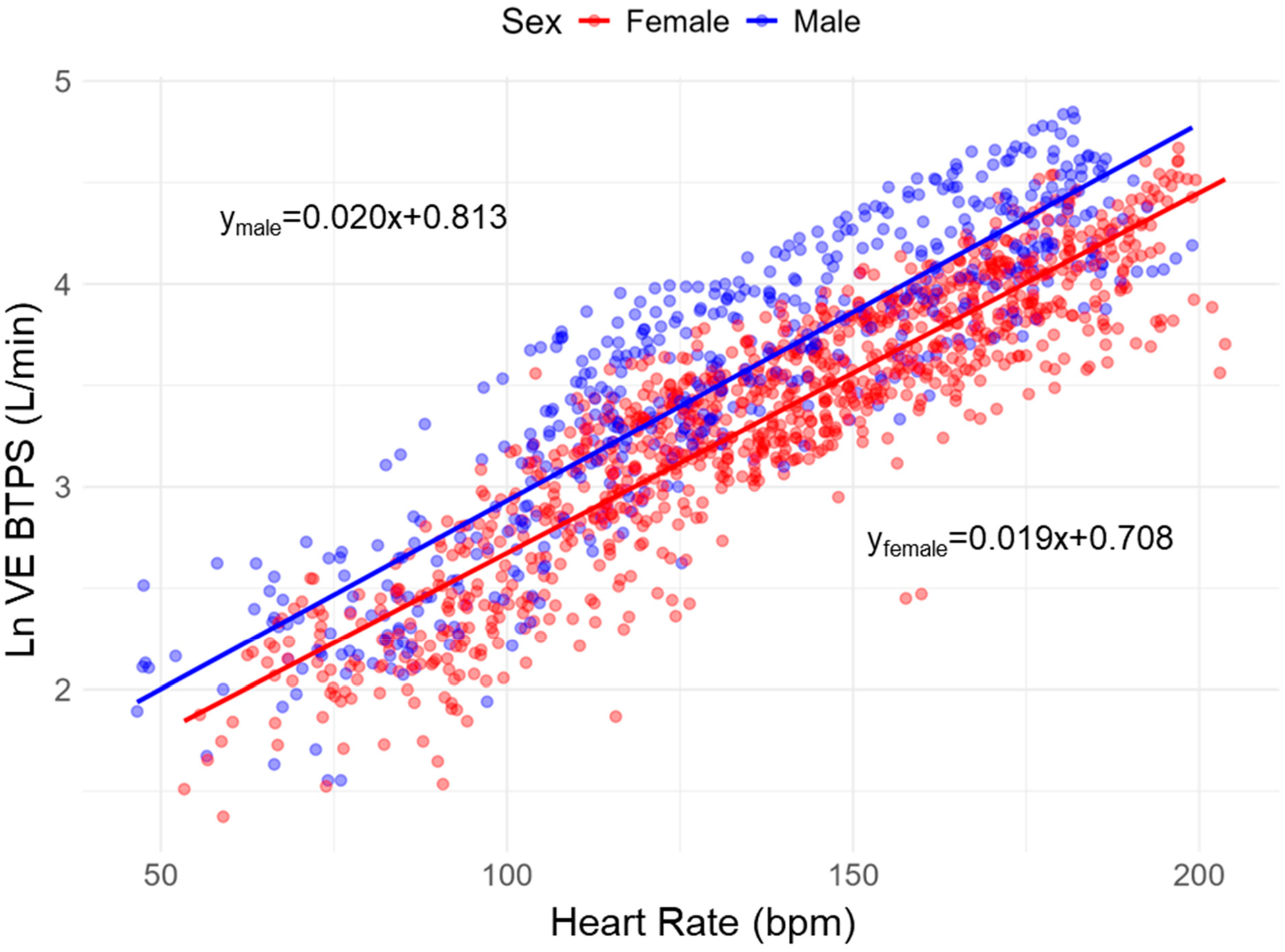
Relationship between minute ventilation (VE) and heart rate (HR) during cardiopulmonary exercise testing (CPET), with patients stratified by sex. Symbols indicate males (*n* = 27, blue) and females (*n* = 14, red). Each point represents an individual measurement, illustrating a positive correlation between lnVE BTPS and HR.

In the fully adjusted model with all covariates, heart rate was a strong predictor of minute ventilation (*p*-value < 0.001). Sex was also significant (*p*-value = 0.003) whereas other covariates including age, race, ethnicity, and BMI were not significantly associated with minute ventilation (*p* > 0.05 for all). Therefore, only sex was explored further in stratified analyses. In our sex-stratified model, males had a slightly higher predicted VE than females for a given HR, reflected by the higher slope (0.020 vs. 0.019) and intercept (0.813 vs. 0.708) (Figure 1). In the interaction model, a significant interaction between sex and heart rate (HR × sex) on minute ventilation was observed (p_interaction_ = 0.047), confirming effect modification by sex. The pseudo-R^2^ value was 0.80 for male stratum and 0.82 for female stratum, indicating good model fit.

When compared to previously published HR-based predictive models of VE, our sex-stratified equations demonstrated the lowest mean percent difference between measured and predicted VE (Table 2). For males, the mean difference was −1.6% (SD 33.0), and for females it was 2.3% (SD 31.9), indicating close alignment between observed and predicted values. In contrast, applying other published equations yielded poor fit to our dataset. For example, the Guo et al. [5] model substantially underestimated VE when applied to our dataset, with mean differences of −92.8% (SD 6.1) in males and −82.6% (SD 9.8) in females. Similarly, the Cruz et al. [6] model underestimated VE in males with a mean percent difference of −59.4% (SD 54.6), while the Cozza et al. [15] models overestimated VE, with mean percent differences of 49.3% (SD 61.1) and 29.8% (SD 53.1) under high and low air pollution conditions, respectively. The Zuurbier et al. [10] model also overestimated VE, with differences of 40.0% (SD 47.9) in men and 56.8% (SD 56.9) in women.

## 4. Discussion

In this study, we present an equation that was developed using predictive modeling to estimate VE for a given HR in a racially and ethnically diverse group of adolescents aged 14–18. Sex was identified as an important contributor to the relationship between HR and VE, and thus, we report sex-specific model estimates. Specifically, males had a slightly higher predicted VE than females for a given HR, suggesting underlying physiological differences in ventilatory responses to exercise. Age, BMI, race, and ethnicity did not significantly affect the relationship between HR and VE when included in the model, and thus, were not included in our final equations. In our comparative analysis, our predictive model had the lowest absolute mean percent difference compared to other exponential models when applied to our dataset, indicating that our equations are the best predictors for our diverse population of adolescents.

Notably, there is a burgeoning field of research exploring the feasibility of using wearable devices that measure respiratory rate (RR) and VE in real-world settings. Bioimpedance chest-patches have shown high accuracy in measuring RR in both static and dynamic activities and in both indoors and outdoors settings [16]. Wearable strain sensors and smart T-shirts have also demonstrated utility in calculating respiration volume and RR [17,18], while recent developments in wearable mask devices allow for the non-invasive, accurate tracking of RR and VE regardless of body movement or breathing route [19,20]. Despite these advances, several barriers limit the widespread implementation of these wearable devices. Technical challenges, including motion artifacts, sensor calibration requirements, and inconsistent signal quality, can compromise measurement accuracy [16,18,21,22]. Anatomical differences across participants affect sensor placement and performance, necessitating patient-specific calibration and large-scale validation [18,22]. Furthermore, cost and poor user compliance due to device bulkiness and potential discomfort during prolonged use (particularly with wearable masks) remain important concerns [20,21,23]. These barriers underscore the importance of developing equations that can predict VE from HR, since HR is a variable that can easily be measured using low-cost, non-invasive, and non-bulky wearables, such as watches or arm bands [8,9].

Our emphasis on HR-based modeling aligns with prior studies conducted in adult populations that have consistently shown strong linear relationships between HR and VE, supporting the use of HR as a surrogate for VE in both laboratory and field settings. Samet et al. and Mermier et al. demonstrated that HR and VE were highly correlated across a range of exercise types and intensities, reinforcing the feasibility of utilizing HR-based estimation of VE when direct VE measurements are difficult to obtain [24,25]. Similarly, Zuurbier et al. showed that HR could be used to estimate VE in real-world settings in an adult study that examined associations between HR and VE while commuting by bus, car, and bicycle [10]. While these studies provide valuable insights into the HR-VE relationship, they primarily focus on adult populations. Children and adolescents may exhibit different patterns due to age-related physiological differences such as higher breathing frequency and ventilation per mass [26].

The relationship between HR and VE has also been studied in younger populations, albeit in a limited fashion. Several studies have investigated the HR-VE relationship in mixed age groups that include children, adolescents, and adults [5,24,25]. However, there is only one prior study in the literature that examines the HR-VE relationship in an adolescent-specific age group. Greenwald et al. (2016) recruited 15 physically active, healthy adolescents aged 15–18 to undergo non-CPET treadmill tests and spirometry [2]. They then created a general linear mixed model (GLMM) that normalized VE by forced vital capacity (FVC) and found HR and breathing rate to be strong predictors of VE. A pooled data analysis conducted by Greenwald et al. in 2019 that included individuals ages 4–80 found age to be an important predictor in the HR-VE relationship as well [27]. Thus, both the Greenwald 2016 study and the pooled data analysis study underscore the benefit of creating predictive models that specifically assess the adolescent age group [2,27]. In a larger cohort of 41 adolescents, our equations predicted VE from HR with high accuracy compared to laboratory-measured VE values obtained using a standardized CPET protocol. Our GEE model focused on population-averaged (marginal) effects, which aligns with our goal of developing a model that is broadly applicable to adolescents and can be used to assess public health questions in larger samples of adolescents.

Importantly, our cohort included a more diverse sample than in prior studies with 19.5% identifying as Hispanic and 7.3% identifying as Black [2,5]. Many studies do not list the race/ethnicity of their participants and thus have not conducted data analyses examining race and/or ethnicity as a predictor [6,10,15,24,25]. Although race and ethnicity did not impact our equations in a statistically significant way, the inclusion of a more diverse population strengthens the ability of the model to detect race/ethnicity-specific effects, improves confidence in the model’s generalizability to urban, multi-ethnic groups, and highlights the importance of ensuring that predictive equations are not biased towards a single demographic.

The statistically significant effect of sex on the HR-VE relationship is consistent with findings from previous studies [5,10,27]. In our model, we found a sex-specific difference in how HR influences VE (*p*-value for interaction= 0.047): for males, the regression line had a slope of 0.020 with an intercept of 0.813, whereas for females, the slope was 0.019 with an intercept of 0.708. Thus, males demonstrated a higher predicted VE than females for a given HR, which is consistent with the anatomical and physiological differences that emerge during puberty. Throughout puberty and into late adolescence, males develop larger lungs, larger central conducting airways, and greater airway luminal area, which reduces airway resistance and allows for greater increases in tidal volume rather than relying on higher respiratory rates [28]. The muscle-fat ratio and testosterone in males further reduce airway resistance, enhancing ventilatory efficiency during exercise [29]. In contrast, females have smaller lung volumes and airway diameters, resulting in higher resistive work of breathing for intense exercise and higher respiratory rates [30–32]. Morphometric and computational airway models demonstrate that airway caliber and lung geometry vary systematically by sex and developmental stage, influencing airflow resistance and particle deposition dynamics [33–35]. After approximately age 14, males develop proportionally larger central airways relative to lung volume than females—a phenomenon known as dysanapsis—which results in greater intrinsic airway resistance in females at equivalent ventilation rates [36]. These distinctions alter the efficiency and metabolic cost of ventilation, providing a mechanistic basis for the sex- and age-specific variability observed in HR–VE relationships. Collectively, these findings support the necessity of sex-stratified and adolescent-specific equations to more accurately capture youth ventilatory responses.

In the comparative analysis, our results suggest that our sex-stratified model provides more accurate predictions of VE for our sample of adolescents compared to previously reported models. Prior HR–VE relationships have largely been derived from adult samples and differ in both structure and context. Guo et al. (2020) used a simple linear regression between HR and VE in adults, whereas Cruz et al. (2020) incorporated workload as an additional predictor [5,6]. Cozza et al. (2015) accounted for air pollution exposure, and Zuurbier et al. (2009) used a polynomial model specific to cycling [10,15]. Thus, the empirical equations generated from these studies, while informative, were parameterized for distinct activity types and primarily adult populations, which likely explains their poor fit to our youth data. Geographic variation may also partially explain why other models’ equations are a poor fit for our sample, as altitude influences the partial pressure of oxygen, which alters minute ventilation. Of note, Greenwald et al. (2016) was not included in our analysis because their predictive equation incorporated FVC [2]. Overall, these findings suggest that an adolescent-specific equation may be necessary to accurately predict VE. Testing our sex-stratified models in another adolescent cohort represents an important next step to evaluate the accuracy and applicability of our model beyond the current sample.

Our equations could be applied in future research to estimate the inhaled dose of air pollutants most strongly associated with asthma development and impaired lung function, such as fine particulate matter (PM_2.5_) and nitrogen oxides [37,38]. According to the U.S. Environmental Protection Agency (EPA), inhaled “dose” of pollution can be defined at several levels [39]. Our equations are particularly suited to estimating the potential exposure dose, the amount of contaminant inhaled through the mouth or nose but not necessarily absorbed, when combined with real-time pollutant exposure data. The internal dose, or amount absorbed into the bloodstream, depends on additional factors such as particle size (ultrafine vs. coarse), chemical composition (gaseous vs. solid), pollutant-specific absorption efficiencies, airflow turbulence and velocity [40,41], and individual respiratory mechanics (i.e., normal vs. asthmatic airways). Prior work has shown that while most inhaled particles deposit in the upper airways, a greater fraction of total inhaled particulate matter tends to reach the distal lung regions in children compared with adults [42]. Thus, incorporating HR-derived VE estimates into exposure models offers a feasible method for estimating potential inhaled exposure dose but does not account for internal dose, which presents a possible future direction for further research.

Our study has several limitations. First, our sample size (*n* = 41) was modest, which may pose a risk for increased Type 1 error. However, the study included a total of 1637 paired HR-VE observations across all participants, providing sufficient data for robust estimation of population-average effects. Our GEE model was fitted with robust (sandwich) standard errors, which help account for potential misspecification of correlation structure. Second, while GEE may obscure individual-level variability compared to GLMM, it was chosen to derive a population-average prediction model suitable for epidemiologic applications rather than individual-level random effects. Third, our equations were developed with HR obtained through CPET, and thus these equations may not be as accurate at the extremes of physical activity when HR doubles or triples in response to rigorous activity. Fourth, the data in our retrospective analysis was derived from adolescents who were initially referred to the CPET lab for suspicion of respiratory illness with concerns of dyspnea, chest pain, asthma, or palpitations. To address this, we restricted our analyses to those with normal CPET values, ensuring that included patients had well-functioning cardiopulmonary systems. However, this restriction excludes adolescents with impaired cardiopulmonary function, a population that is both clinically and environmentally relevant. This introduces referral bias and limits the generalizability of our model, necessitating external validation of our model in a larger, more diverse adolescent population. Additionally, other physiological and behavioral factors, including training status, athleticism, stress, and anxiety, can influence HR and VE and should be considered in future validation studies [43,44]. Finally, a large proportion of participants declined to answer the race and ethnicity questions (31.7% for race, 29.3% for ethnicity) or selected “Other” (22%). Declined responses may reflect participant confusion, discomfort, or concerns about confidentiality, while “Other” responses may indicate the absence of categories that reflect how participants view their identities. These limitations could lead to misclassification or underrepresentation of certain groups, potentially obscuring differences in cardiopulmonary function or pollutant exposure across racial and ethnic populations. Going forward, our predictive equations require external validation in larger, more racially/ethnically and physiologically diverse adolescent cohorts.

## 5. Conclusions

In this preliminary study, we confirmed that heart rate is a strong predictor of minute ventilation in adolescents and found that sex significantly modifies this relationship. By using a linear regression model to develop sex-stratified equations, we achieved closer alignment between measured and predicted VE values than previously published HR–VE models derived from adult or geographically distinct populations. These findings are consistent with prior research demonstrating a robust HR–VE association while extending its applicability to an adolescent-only cohort, a population in which accurate exposure modeling has been limited.

From an environmental health perspective, the ability to predict VE from HR provides a valuable tool for estimating inhaled pollutant exposure in real-world settings when direct measurement of VE is not possible. As a pilot analysis, this work offers foundational HR–VE equations that could be further validated in different samples of adolescents. Ultimately, these equations may be used to support studies assessing personal environmental health risks in active youth and to inform targeted public health interventions to mitigate these risks.

## Figures and Tables

**Table 1.** Demographics Table for Study Participants.

Variable	Value (Percentage or Standard Deviation; Range)
Sex	Female: 14 (34.1%)
	Male: 27 (65.9%)
Race	Black: 3 (7.3%)
	White: 16 (39.0%)
	Other: 9 (22%)
	Declined: 13 (31.7%)
Ethnicity	Hispanic: 8 (19.5%)
	Non-Hispanic: 21 (51.2%)
	Declined: 12 (29.3%)
Mean Age (years)	15.4 (1.2; 14−18)
Mean Height (cm)	165.9 (8.8; 149−180)
Mean Weight (kg)	60.6 (10.4; 45−90)
Mean BMI (kg/m^2^)	22.0 (2.5; 19−28)

**Table 2.** Mean percent difference between measured minute ventilation (VE) and predicted VE from our model versus other published HR-based predictive models.

Reference	Population	Sex (Number)	Model Equation	Average % Difference (Standard Deviation) Between Measured and Predicted VE
Current study	New York City, United States Ages 14–18	Male (*n* = 27)	Ln(VE) = 0.020 × HR + 0.813	−1.6% (33.0)
		Female (*n* = 14)	Ln(VE) = 0.019 × HR + 0.708	2.3% (31.9)
Guo et al., 2020 [5]	Beijing, China Ages 16–21	Male (*n* = 42)	Ln(VE) = 0.007 × HR + 0.559	−92.8% (6.1)
		Female (*n* = 38)	Ln(VE) = 0.006 × HR + 0.647	−82.6% (9.8)
Cruz et al., 2020 [6]	Sao Paulo, Brazil Males (mean age 27.8 ± 5.4 years, range not specified)	Male (*n* = 18)	Ln(VE) = 0.021 × HR + 1.16	−59.4% (54.6)
Cozza et al., 2015 [15]	Sao Paulo, Brazil Males (ages 18–65)	Male (*n* = 33; high air pollution)	Ln(VE) = 0.025 × HR + 0.54	49.3% (61.1)
		Male (*n* = 17; low air pollution)	Ln(VE) = 0.025 × HR + 0.40	29.8% (53.1)
Zuurbier et al., 2009 [10]	Arnhem, The Netherlands Ages 18–56	Male (*n* = 10)	Ln(VE) = 0.021 × HR + 1.03	40.0% (47.9)
		Female (*n* = 24)	Ln(VE) = 0.023 × HR + 0.57	56.8% (56.9)

## Data Availability

Data supporting the results of this study can be made available upon request to the corresponding author.

## References

[1] Gauderman WJ; Avol E; Gilliland F; Vora H; Thomas D; Berhane K; McConnell R; Kuenzli N; Lurmann F; Rappaport E; The effect of air pollution on lung development from 10 to 18 years of age. N. Engl. J. Med 2004, 351, 1057–1067. 15356303 10.1056/NEJMoa040610

[2] Greenwald R; Hayat MJ; Barton J; Lopukhin A A Novel Method for Quantifying the Inhaled Dose of Air Pollutants Based on Heart Rate, Breathing Rate and Forced Vital Capacity. PLoS ONE 2016, 11, e0147578. 26809066 10.1371/journal.pone.0147578 PMC4726691

[3] Daigle CC; Chalupa DC; Gibb FR; Morrow PE; Oberdörster G; Utell MJ; Frampton MW Ultrafine particle deposition in humans during rest and exercise. Inhal. Toxicol 2003, 15, 539–552. 12692730 10.1080/08958370304468

[4] Tran D Cardiopulmonary Exercise Testing. Methods Mol. Biol 2018, 1735, 285–295. 29380321 10.1007/978-1-4939-7614-0_18

[5] Guo Q; Zhao Y; Shao J; Cao S; Wang Q; Wu W; Duan X Using heart rate to estimate the minute ventilation and inhaled load of air pollutants. Sci. Total Environ 2021, 763, 143011. 33138987 10.1016/j.scitotenv.2020.143011

[6] Cruz R; Alves DL; Rumenig E; Gonçalves R; Degaki E; Pasqua L; Koch S; Lima-Silva AE; Koehle MS; Bertuzzi R Estimation of minute ventilation by heart rate for field exercise studies. Sci. Rep 2020, 10, 1423. 31996732 10.1038/s41598-020-58253-7 PMC6989498

[7] Vitazkova D; Foltan E; Kosnacova H; Micjan M; Donoval M; Kuzma A; Kopani M; Vavrinsky E Advances in Respiratory Monitoring: A Comprehensive Review of Wearable and Remote Technologies. Biosensors 2024, 14, 90. 38392009 10.3390/bios14020090 PMC10886711

[8] Nelson BW; Low CA; Jacobson N; Areán P; Torous J; Allen NB Guidelines for wrist-worn consumer wearable assessment of heart rate in biobehavioral research. npj Digit. Med 2020, 3, 90. 32613085 10.1038/s41746-020-0297-4 PMC7320189

[9] Schweizer T; Gilgen-Ammann R Wrist-Worn and Arm-Worn Wearables for Monitoring Heart Rate During Sedentary and Light-to-Vigorous Physical Activities: Device Validation Study. JMIR Cardio 2025, 9, e67110. 40116771 10.2196/67110 PMC11951816

[10] Zuurbier M; Hoek G; van den Hazel P; Brunekreef B Minute ventilation of cyclists, car and bus passengers: An experimental study. Environ. Health 2009, 8, 48. 19860870 10.1186/1476-069X-8-48 PMC2772854

[11] Hesse A; White M; Lundstrom C The prevalence of gas exchange data processing methods: A semi-automated scoping review. Int. J. Sports Med 2025, 46, 227–236. 39832763 10.1055/a-2495-5364

[12] Bruce RA Exercise testing of patients with coronary heart disease. Principles and normal standards for evaluation. Ann. Clin. Res 1971, 3, 323–332. 5156892

[13] Washington RL; Bricker JT; Alpert BS; Daniels SR; Deckelbaum RJ; Fisher EA; Gidding SS; Isabel-Jones J; E Kavey R; Marx GR Guidelines for exercise testing in the pediatric age group. From the Committee on Atherosclerosis and Hypertension in Children, Council on Cardiovascular Disease in the Young, the American Heart Association. Circulation 1994, 90, 2166–2179. 7923708 10.1161/01.cir.90.4.2166

[14] Buehler S; Lozano-Zahonero S; Schumann S; Guttmann J Monitoring of intratidal lung mechanics: A Graphical User Interface for a model-based decision support system for PEEP-titration in mechanical ventilation. J. Clin. Monit. Comput 2014, 28, 613–623. 24549460 10.1007/s10877-014-9562-x

[15] Cozza IC; Zanetta DMT; Fernandes FLA; da Rocha FMM; de Andre PA; Garcia MLB; Paceli RB; Prado GF; Terra-Filho M; Saldiva PHDN; An approach to using heart rate monitoring to estimate the ventilation and load of air pollution exposure. Sci. Total Environ 2015, 520, 160–167. 25813969 10.1016/j.scitotenv.2015.03.049

[16] Qiu C; Wu F; Han W; Yuce MR A Wearable Bioimpedance Chest Patch for Real-Time Ambulatory Respiratory Monitoring. IEEE Trans. Biomed. Eng 2022, 69, 2970–2981. 35275808 10.1109/TBME.2022.3158544

[17] Chu M; Nguyen T; Pandey V; Zhou Y; Pham HN; Bar-Yoseph R; Radom-Aizik S; Jain R; Cooper DM; Khine M Respiration rate and volume measurements using wearable strain sensors. npj Digital Med. 2019, 2, 8. 10.1038/s41746-019-0083-3 PMC6550208 31304358

[18] Romano C; Lo Presti D; Silvestri S; Schena E; Massaroni C Flexible Textile Sensors-Based Smart T-Shirt for Respiratory Monitoring: Design, Development, and Preliminary Validation. Sensors 2024, 24, 2018. 38544279 10.3390/s24062018 PMC10974106

[19] Tipparaju VV; Wang D; Yu J; Chen F; Tsow F; Forzani E; Tao N; Xian X Respiration pattern recognition by wearable mask device. Biosens. Bioelectron 2020, 169, 112590. 32927349 10.1016/j.bios.2020.112590 PMC7572779

[20] Xu Y; Li Q; Tang Z; Liu J; Xiang B Towards Accurate, Cost-Effective, Ultra-Low-Power and Non-Invasive Respiration Monitoring: A Reusable Wireless Wearable Sensor for an Off-the-Shelf KN95 Mask. Sensors 2021, 21, 6698. 34695911 10.3390/s21206698 PMC8540598

[21] Fakhrulddin SS; Bhatt V Wearable biosensors and devices for lung function monitoring. Prog. Mol. Biol. Transl. Sci 2025, 215, 355–384. 40683748 10.1016/bs.pmbts.2025.05.007

[22] Sato H; Nagano T; Izumi S; Yamada J; Hazama D; Katsurada N; Yamamoto M; Tachihara M; Nishimura Y; Kobayashi K Prospective observational study of 2 wearable strain sensors for measuring the respiratory rate. Medicine 2024, 103, e38818. 39029069 10.1097/MD.0000000000038818 PMC11398755

[23] Althobiani MA; Khan B; Shah AJ; Ranjan Y; Mendes RG; Folarin A; Mandal S; Porter JC; Hurst JR Clinicians’ Perspectives of Wearable Technology to Detect and Monitor Exacerbations of Chronic Obstructive Pulmonary Disease: Mixed-Method Survey. Int. J. Chronic Obstr. Pulm. Dis 2023, 18, 1401–1412. 10.2147/COPD.S405386 PMC10349580 37456915

[24] Samet JM; Lambert WE; James DS; Mermier CM; Chick TW Assessment of heart rate as a predictor of ventilation. Res. Rep. Health Eff. Inst 1993, 59, 19–55; discussion 57–69. 8216970

[25] Mermier CM; Samet JM; Lambert WE; Chick TW Evaluation of the relationship between heart rate and ventilation for epidemiologic studies. Arch. Environ. Health 1993, 48, 263–269. 8357278 10.1080/00039896.1993.9940371

[26] Gratas-Delamarche A; Mercier J; Ramonatxo M; Dassonville J; Préfaut C Ventilatory response of prepubertal boys and adults to carbon dioxide at rest and during exercise. Eur. J. Appl. Physiol. Occup. Physiol 1993, 66, 25–30. 8425509 10.1007/BF00863395

[27] Greenwald R; Hayat MJ; Dons E; Giles L; Villar R; Jakovljevic DG; Good N Estimating minute ventilation and air pollution inhaled dose using heart rate, breath frequency, age, sex and forced vital capacity: A pooled-data analysis. PLoS ONE 2019, 14, e0218673. 31287820 10.1371/journal.pone.0218673 PMC6615621

[28] Nève V; Girard F; Flahault A; Boulé M Lung and thorax development during adolescence: Relationship with pubertal status. Eur. Respir. J. Eur. Respir. Soc 2002, 20, 1292–1298. 10.1183/09031936.02.00208102 12449187

[29] Kim JH; Kim JA; Ha EK; Jee HM; Lee SW; Jung MK; Lee S; Shin YH; Yoo E-G; Han MY Sex differences in body composition affect total airway resistance during puberty. BMC Pediatr. 2022, 22, 143. 35300646 10.1186/s12887-022-03198-1 PMC8928689

[30] Mann LM; Angus SA; Doherty CJ; Dominelli PB Evaluation of sex-based differences in airway size and the physiological implications. Eur. J. Appl. Physiol 2021, 121, 2957–2966. 34331574 10.1007/s00421-021-04778-2

[31] Harms CA Does gender affect pulmonary function and exercise capacity? Respir. Physiol. Neurobiol 2006, 151, 124–131. 16406740 10.1016/j.resp.2005.10.010

[32] Peters CM; Leahy MG; Hohert G; Lane P; Lam S; Sin DD; McKenzie DC; Sheel AW Airway luminal area and the resistive work of breathing during exercise in healthy young females and males. J. Appl. Physiol. (1985) 2021, 131, 1750–1761. 34709072 10.1152/japplphysiol.00418.2021

[33] Christou S; Chatziathanasiou T; Angeli S; Koullapis P; Stylianou F; Sznitman J; Guo HH; Kassinos SC Anatomical variability in the upper tracheobronchial tree: Sex-based differences and implications for personalized inhalation therapies. J. Appl. Physiol. (1985) 2021, 130, 678–707. 33180641 10.1152/japplphysiol.00144.2020

[34] Worth Longest P; Hindle M; Das Choudhuri S Effects of generation time on spray aerosol transport and deposition in models of the mouth-throat geometry. J. Aerosol Med. Pulm. Drug Deliv 2009, 22, 67–83. 18956949 10.1089/jamp.2008.0692

[35] Jin G; Kumar H; Clark AR; Burrowes KS; Hoffman EA; Tawhai MH Evaluating the role of sex-related structure-function differences on airway aerosol transport and deposition. J. Appl. Physiol. (1985) 2024, 137, 1285–1300. 39169840 10.1152/japplphysiol.00898.2023 PMC11918303

[36] Ripoll JG; Guo W; Andersen KJ; Baker SE; Wiggins CC; Shepherd JRA; Carter RE; Welch BT; Joyner MJ; Dominelli PB Sex differences in paediatric airway anatomy. Exp. Physiol 2020, 105, 721–731. 32003484 10.1113/EP088370 PMC7641092

[37] Zhang W; Ma R; Wang Y; Jiang N; Zhang Y; Li T The relationship between particulate matter and lung function of children: A systematic review and meta-analysis. Environ. Pollut 2022, 309, 119735. 35810981 10.1016/j.envpol.2022.119735

[38] Brumberg HL; Karr CJ; Council on Environmental Health. Ambient Air Pollution: Health Hazards to Children. Pediatrics 2021, 147, e2021051484. 34001642 10.1542/peds.2021-051484

[39] US EPA. Exposure Assessment Tools by Routes—Inhalation. Available online: https://www.epa.gov/expobox/exposure-assessment-tools-routes-inhalation (accessed on 5 November 2025).

[40] Sommerfeld M; Sgrott OL; Taborda MA; Koullapis P; Bauer K; Kassinos S Analysis of flow field and turbulence predictions in a lung model applying RANS and implications for particle deposition. Eur. J. Pharm. Sci 2021, 166, 105959. 34324962 10.1016/j.ejps.2021.105959

[41] Liu H; Ma S; Hu T; Ma D Computational investigation of flow characteristics and particle deposition patterns in a realistic human airway model under different breathing conditions. Respir. Physiol. Neurobiol 2023, 314, 104085. 37276915 10.1016/j.resp.2023.104085

[42] Voliotis A; Bezantakos S; Besis A; Shao Y; Samara C Mass dose rates of particle-bound organic pollutants in the human respiratory tract: Implications for inhalation exposure and risk estimations. Int. J. Hyg. Environ. Health 2021, 234, 113710. 33618174 10.1016/j.ijheh.2021.113710

[43] Reimers AK; Knapp G; Reimers C-D Effects of Exercise on the Resting Heart Rate: A Systematic Review and Meta-Analysis of Interventional Studies. J. Clin. Med 2018, 7, 503. 30513777 10.3390/jcm7120503 PMC6306777

[44] Javorka M; El-Hamad F; Czippelova B; Turianikova Z; Krohova J; Lazarova Z; Baumert M Role of respiration in the cardiovascular response to orthostatic and mental stress. American Journal of Physiology-Regulatory, Integrative and Comparative Physiology. Am. Physiol. Soc 2018, 314, R761–R769. 10.1152/ajpregu.00430.2017 29443551

